# Exploring the Effect of Collective Cultural Attributes on Covid-19-Related Public Health Outcomes

**DOI:** 10.3389/fpsyg.2021.627669

**Published:** 2021-03-23

**Authors:** Aysegul Erman, Mike Medeiros

**Affiliations:** ^1^Toronto General Hospital Research Institute, University Health Network, Toronto, ON, Canada; ^2^Toronto Health Economics and Technology Assessment Collaborative (THETA) Collaborative, University Health Network, University of Toronto, Toronto, ON, Canada; ^3^Department of Political Science, Faculty of Social and Behavioral Sciences, University of Amsterdam, Amsterdam, Netherlands

**Keywords:** COVID-19, public health, Hofstede cultural dimensions, meta-regression, culture, pandemic, meta-analaysis, health services

## Abstract

Infections and deaths associated with COVID-19 show a high degree of heterogeneity across different populations. A thorough understanding of population-level predictors of such outcomes is crucial for devising better-targeted and more appropriate public health preparedness measures. While demographic, economic, and health-system capacity have featured prominently in recent work, cultural, and behavioral characteristics have largely been overlooked. However, cultural differences shape both the public policy response and individuals' behavioral responses to the crisis in ways that can impact infection dynamics and key health outcomes. To address this gap, we used meta-analytic methods to explore the global variability of three public health outcomes (i.e., crude test positivity, case/infection fatality, and mortality risk) during the first wave of the pandemic. This set of analyses identified several cultural/behavioral attributes (e.g., uncertainty avoidance and long-term vs. short-term normative orientation) as independent predictors of public health outcomes after adjusting for key demographic, political, economic, and health-system-related predictors; which were robust in sensitivity analyses. In conclusion, this study clearly demonstrates that cultural attributes do in fact account for some of the global disparities in COVID-19-attributed health outcomes. As a consequence, policymakers should more explicitly consider a society's cultural attributes alongside other important parameters such as demographic characteristics and health system constraints in order to develop better tailored and more effective policy responses.

## Introduction

The exceptional global phenomenon of the COVID-19 crisis has led to a situation where societies that vary considerably—in terms of social and cultural values as well as economic and demographic characteristics—found themselves having to deal with a common public health emergency simultaneously, with a variable degree of success in mitigating infections and infection-related fatalities.

To mitigate the sudden surge in the number of COVID-19 cases in the early weeks of March 2020, many countries have implemented large-scale social distancing measures to varying degrees, with the aim of reducing the transmission of SARS-CoV-2 (Koo et al., 2020; Mahase, 2020). During this time, which comprises the first wave of the pandemic, countries have also expanded testing for SARS-CoV-2 in combination with contact tracing and isolation to varying extents. With many nations reporting a reduction in both incident cases and deaths, a gradual relaxation of confinement commenced in early June. However, with the resurgence of a second wave in October, restrictions were once again rapidly reintroduced in many settings. Despite the apparently similar initial reactions to the pandemic, different nations have at times taken quite divergent approaches to manage the crisis; differing with respect to scope, scale and implementation (Yan et al., 2020). Moreover, the attitudes of the general population toward the crisis at large and the public compliance with behavioral recommendations also exhibit a considerable degree of variation (Sabat et al., 2020).

Unsurprisingly, both the infection dynamics and fatalities associated with COVID-19 are extremely heterogeneous across different countries and populations. Based on available patient-level data, risk of severe illness, and death are typically highest among older adults (>65 years), as well as immunocompromised individuals, and those with comorbid conditions (Onder et al., 2020). Moreover, COVID-19-attrributed mortality also appears to rise rapidly as the surge in the number of severe cases requiring specialized care exceed existing health system capacity (Armocida et al., 2020; Onder et al., 2020). In particular, health system constraints in terms of the number of healthcare workers, hospital beds, contact tracing, and testing capacity, as well as the availability of personal protective equipment have been a global concern in the fight against COVID-19.

However, while public health capacity, demographic differences and socioeconomic development are certainly important factors that can account for such disparities, cultural characteristics should not be overlooked. Culture has essentially been understood as a set of norms or common values shared by a defined group of individuals (Lehman et al., 2004). Cultural factors have consistently been shown to either directly affect or moderate a large variety of behavioral phenomenon (Schneider and De Meyer, 1991; Borg, 2014a; Bernhardsdóttir, 2015; Venkateswaran and George, 2020).

Given the role that cultural norms play in society at large, it is reasonable to expect that various cultural attributes can influence the outcomes of a pandemic, as such outcomes are dependant on social compliance to broad and varied behavioral strategies. Behavior modification is an important aspect of public policy as it “almost always attempts to get people to do things they otherwise would not have done, or it enables them to do things they might not have done otherwise” (Schneider and Ingram, 1990). Inducing citizens to comply with laws and policies is therefore a goal of policymakers. Such objectives can be imperative in public health crises. While social norms can guide citizens to act in a socially appropriate way (Morris et al., 2015), cultural distinctions can nevertheless impact the manner in which encouraged socially conscious behaviors are adopted by individuals (Nash et al., 2019). However, the extent to which such differences in sociocultural norms may influence important outcomes during such a health crisis has not yet been thoroughly explored.

In this study, we address this scholarly gap by exploring the variation in the crude test positivity, crude case fatality among confirmed cases of infection and the mortality risk among the population which has been attributed to COVID-19 during the initial phase of the pandemic. Specifically, we examine the extent to which cultural attributes can explain these disparities alongside other key factors (e.g., demographics, health system capacity, timing of the epidemic) at a population-level.

Ultimately, the results of this analysis clearly demonstrate that cultural attributes do in fact account for some of the global disparities in COVID-19-attribtuted public health outcomes. As a consequence, policymakers should consider these cultural attributes alongside demographic characteristics and health system constraints to develop better tailored and more effective policy responses going forward.

## Materials and Methods

### Country Selection and Data Sources

We explored the variation in COVID-19-attributed deaths in 73 countries during the first wave of the pandemic (up to September 20, 2020). Together these 73 countries account to ~93% of confirmed cases and ~96% of deaths which have been directly attributed to COVID-19 during this time period; accounting for a total of 29,540,648 detected infections and 932,491 deaths over an average follow-up time of 213 days (range: 185–294) from diagnosis of the initial case to the time of the analysis.

We collected data on cultural characteristics of countries using the Hofstede (2010) model, a well-accepted and frequently used method for evaluating sociocultural variation between countries (Hofstede, 2010, 2011). Countries were selected based on availability of data on key outcomes during this time frame, as well as the availability of data on cultural characteristics as measured by Hofstede (Hofstede, 2011)^1^_._ The Hofstede model is comprised of six cultural dimensions: (1) individualism vs. collectivism; (2) uncertainty avoidance (i.e., the degree of discomfort with uncertainty); (3) indulgence vs. restraint; (4) long-term vs. short-term normative orientation; (5) power distance (i.e., level of hierarchy within a society); and (6) masculinity vs. femininity. In brief, these six dimensions conceptualize and measure independent preferences for each cultural construct in order to describe the cultural characteristics of each country (Hofstede, 2010, 2011).

Additionally, we collected data on the extent of SARS-CoV-2 testing, the number of confirmed cases of infection, the number of COVID-19-attributed deaths during this time period, as well as the time of first confirmed case and first death in each country using publicly available datasets (Dong et al., 2020; WHO, 2021). We obtained the most recent available data on demographics, health system, and economic indicators using country-level data from the (World Bank, 2021) and the WHO Global Health Observatory Data Repository (WHO, 2016). Finally, political characteristic of nations were collected using the polity data series, a widely used dataset that indicates the level of democracy, anocracy and autocracy in each country by considering electoral processes for political competitiveness and openness, the level of political participation, and the extent of separation of power (i.e., constraints on executive authority) (Marshall and Gurr, 2020). Specifically, for this purpose we use the Combined Polity Score for 2018, the most recent year for which data on the political characteristics (i.e., regime type) of countries were available. All potential exploratory variables were measured before the onset of the pandemic.

### Outcomes

We specifically evaluated three important public health outcomes: (1) crude test positivity rate (as a proxy for disease spread), (2) crude infection or case fatality rate (CFR), and (3) mortality risk. We defined crude test positivity as the number of confirmed cases of infection as a ratio of the total number of tests; crude CFR was defined as the probability of death among all confirmed infections; and mortality risk was defined as the number of COVID-19-attributed deaths per 1,000 population. These metrics measure inter-related but different attributes of the public health burden of COVID-19. We employ the crude test positivity rate as a proxy for the extent of disease spread and CFR and mortality risk as two different measures of fatality. For instance, mortality can be affected by the size of the epidemic among the general population, the underlying demographic composition of a population in terms of risk factors (e.g., elderly population), the health system capacity to cope with a large surge in critical cases, as well as the incidence of concentrated outbreaks among more vulnerable subgroups (i.e., long-term care facilities). Whereas, the crude CFR represents the lethality of the disease among infected individuals and is likely to be affected by similar factors; however, this metric could better reflect an inability to prevent outbreaks of infections among the more vulnerable risk groups within a society (e.g., long-term care homes). Moreover, unlike the mortality risk metric, CFR is also more likely to be affected by the testing strategies employed by different countries; with broader testing identifying more asymptomatic individuals resulting in a lower CFR estimate relative to settings with more restricted testing policies or capacities; this is also likely to be the case for test positivity.

### Meta-Analysis

We used random-effects meta-analysis to first pool outcomes reported by countries during the initial phase of the pandemic (up to September 20, 2020). A random-effect model was chosen to account for the variability across estimates derived from heterogeneous settings, populations, and contexts. In this approach observations with a greater precision (i.e., smaller variance) are weighted more relative to observations with less precision following a logit transformation to stabilize the variance of proportions whereas observations that deviate more from the pooled mean receive a lower weight (Barendregt et al., 2013; Schwarzer et al., 2019). Confidence and prediction intervals were generated for all pooled estimates to reflect the uncertainty and the distribution of expected range of true estimates in a similar set of observations (IntHout et al., 2016).

### Meta-Regression Models

The independent effects that the collective cultural attributes of countries may have had on the observed COVID-19 attributed public health outcomes during this timeframe were explored using random-effects meta-regression models that control for a range of important confounders and that account for the variability in reported outcomes. To explore the effect of cultural attributes on these fatality outcomes, two different model specification approaches were employed: a theory-driven *a priori* variable selection approach and an exploratory statistical model specification using bootstrap variable selection approach (Austin and Tu, 2004).

The *a priori* model was developed using a theory-driven approach to specifically investigate the effect of two cultural/behavioral dimensions that most frequently explain variation in crisis management and/or public health practice based on the literature: individualism and uncertainty avoidance (i.e., the level of discomfort with uncertain situations) (Deschepper et al., 2008; Borg, 2014a; Verma et al., 2016; Masood et al., 2019). Specifically, these two cultural constructs have previously been linked to a variety of factors, which can impact pandemic-related outcomes. For instance, individualist and collectivist societies have been shown to have different attitudes and practices in terms of eldercare and other social norms (Pyke and Bengtson, 1996). Similarly, uncertainty avoidance has been associated with differences in medical practice (e.g., suboptimal communication with patients, inappropriate antibiotic use), as well as with negative health consequences (e.g., prevalence of antimicrobial resistant pathogens) (Meeuwesen et al., 2009; Smith, 2015; Stojcic et al., 2016). We therefore expect that countries that score higher on uncertainty avoidance and on individualism to have more negative COVID-19 related outcomes at the population level.

In the *a priori* models we focused on the two most pertinent cultural constructs in order to avoid model overfit (Thompson and Higgins, 2002). The *a priori* models also adjusted for important predetermined predictors such as underlying demographics (e.g., age distribution), indicators of health system capacity (e.g., numbers of healthcare workers and hospital beds, the extent of testing coverage), economic indicators [i.e., gross domestic product (GDP) per capita in 2019], a political indicator (i.e., the polity score, to control for potential variability in reported outcomes that may arise from differences in good governance and accountability), while also controlling for the timing of the outbreak (i.e., days since first death on record). Additionally, as a broader exploratory approach, an alternative model specification process, the bootstrap variable selection method, was used to select potentially important variables from a regression model including a larger set of demographic and sociocultural predictors as described in Table 1.

**Table 1:** Characteristics of countries included in the analysis.

**Country characteristics**	***N***	**Mean (SD)**	**Min**	**Max**
GDP per capita ($US, 2019)	73	25,113 (24,154)	858	114,705
Population density (pop per km^2^)	73	264 (943)	3.2	7,953
Urban population (%)	73	72 (17)	21	100
**Demographics and health**				
Life expectancy at birth (years)	73	78 (4.6)	64.0	84.0
Proportion over 65 years (%)	73	14 (6.2)	3.4	28.0
Proportion over 80 years (%)	73	3.5 (2.0)	0.5	8.7
Elderly dependency ratio (% of adults)	73	22 (9.9)	4.8	47.0
Prevalence of smoking (%)	73	24 (8.8)	4.4	43.0
Prevalence of overweight (% of adults)	73	54 (13)	18.0	70.0
**Health system capacity**				
Hospital beds (*n*. per 1,000 pop)	73	3.6 (2.5)	0.3	13.0
Healthcare workers (*n*. per 1,000 pop)	73	9.7 (5.7)	0.8	23.0
Doctors (*n*. per 1,000 pop)	73	2.8 (1.4)	0.1	7.1
Nurses (*n*. per 1,000 pop)	73	6.9 (4.9)	0.4	19.0
Out-of-pocket health expenditure (%)	73	29 (16)	7.8	74.0
Health expenditure (% of GDP)	73	7.4 (2.7)	1.2	17.0
Pandemic-specific data^*^				
Number of confirmed cases	73	395,125 (1,127,817)	1,068	6,804,814
Number of deaths	73	12,584 (30,974)	10	199,509
Testing coverage (*n*. test per 1 million pop)	73	155,884 (190,681)	1,314	1,253,796
Time since first case (days)	73	213 (20)	185	294
Time since first death (days)	73	188 (15)	163	253
Cultural dimensions^**^				
Individualism vs. collectivism	73	44 (23)	8	91
Uncertainty avoidance	73	69 (22)	8	112
Indulgence vs. restraint	73	47 (22)	0	100
Long-term vs. short-term normative orientation	73	47 (23)	7	100
Masculinity vs. femininity	73	48 (20)	5	110
Power distance index	73	60 (22)	11	104
Political dimensions^***^				
Polity (democracy vs. authoritarianism)	73	6.7 (4.8)	–7.0	10.0

*Characteristics of 73 countries included in the analysis*.

* *Pandemic-related data is collected at the last follow-up date (September 20, 2020)*.

** *Cultural dimensions: higher values reflect a stronger attachment for one cultural dimension relative to its complement (e.g., a higher value on individualism vs. collectivism dimension indicates a stronger preference for individualism relative to collectivism)*.

*** *Polity is a measure of regime type in each country ranging from democracy to authoritarianism*.

In terms of missing data, <1% of the values in the dataset were missing in the original dataset. Missing values were distributed as follows across variables: one value missing (*N* = 7), two missing (*N* = 1), three missing (*N* = 3), and four missing (*N* = 1). Prior to the regression analyses, missing data on predictors were imputed using multivariate imputation by chained equations methods generating 15 imputed datasets using all collected variables contained in the original dataset with 50 iterations per imputation via classification and regression tree method. Model specification was performed on all imputed datasets and outputs were pooled.

Meta-regressions were performed using a logit transformation of the dependant variables to stabilize the variance of proportions (Barendregt et al., 2013; Schwarzer et al., 2019). Each regression coefficient was transformed to odds ratios (OR), whereby an OR >1 indicates a positive association and OR <1 indicates a negative association between the covariate with the outcome. Pseudo R-squared values were used to quantify the proportion of observed variability explained by covariates included in the models. Akaike and Bayesian information criterion (AIC and BIC) were estimated to compare models in terms of model-fit and parsimony.

### Sensitivity Analysis

Influential observations in each model were identified using the leave-one-out diagnostic methods (Viechtbauer, 2010). Models were refitted after omitting influential observations for test positivity (China, Egypt, Singapore Luxemburg, and Jordan), case fatality (Singapore, Luxemburg, and the Philippines), and mortality risk (China, Peru, Vietnam, and Thailand) to evaluate the robustness of findings and to describe how these can impact model specification.

### Extended Analysis

While the main analysis focused on the first wave of the pandemic, in a supplemental analysis we evaluated our models over an extended timeframe that covers the first two waves of the pandemic up to February 12, 2021.

### Statistical Analysis

All statistical analyses were performed using R version 3.6.3 with RStudio. Meta-analyses and Meta-regression analyses were performed with the “metafor” package using a logit transformation to stabilize the variance of proportions (Barendregt et al., 2013; Schwarzer et al., 2019). A *p* < 0.05 was used to signify statistically significant associations.

## Results

### Country Characteristics

The analysis included 73 countries representing a large majority of the confirmed cases of infection (93%) and COVID-19-attributed deaths (96%) worldwide. The cultural, economic, demographic, and pandemic related characteristics of these countries are summarized in Table 1. In brief, the analysis included 35 (48%) countries from Europe and Central Asia; 11 (15%) from the Latin America and Caribbean region; 11 (15%) from the East Asia and Pacific region; 9 (12%) from Middle East and North Africa; 3 (4%) from South Asia; 2 (3%) from North America; and 2 (3%) Sub-Saharan Africa. The average per capita GDP was USD $25,113 (range: $858–$114,705), the proportion of the population aged above 65 years was 14% (range: 3–28%). In terms of health system capacity, on average, for every 1,000 individuals, there were 3.6 hospital beds (range: 0.3–13), 2.8 doctors (range: 0.1–7), and 6.9 nurses (range: 0.4–19). With respect to COVID-19 related factors, there were a total of ~574 million tests performed for SARS-CoV-2 over this first-wave of the pandemic, translating to an average testing coverage of 155,884 tests per 1 million population (range: 1,314–1,253,796).

**Figure 1:**
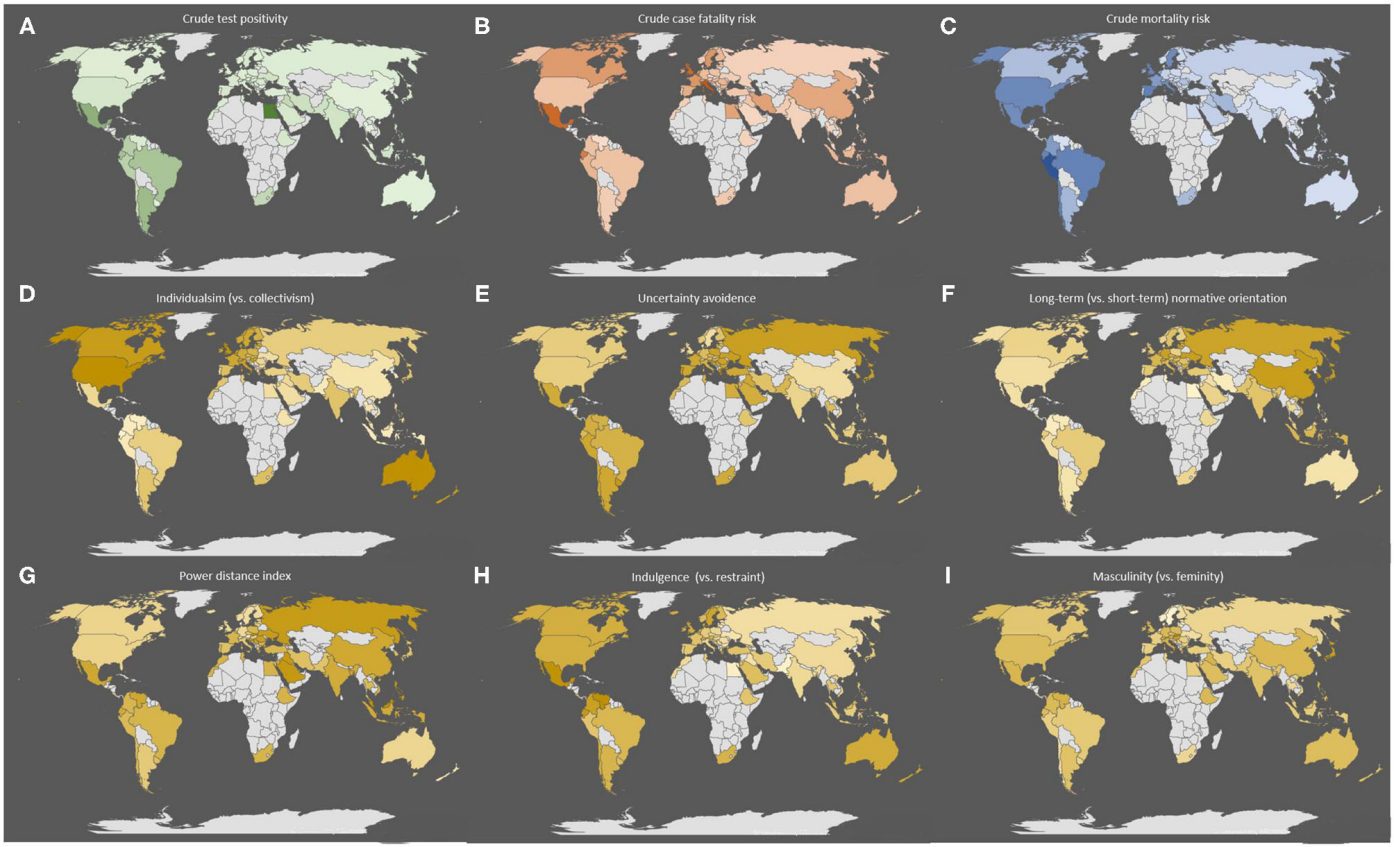
Geographical distribution of the three health outcomes and six cultural attributes across the 73 countries included in the analysis. The figure depicts the geographical distribution of countries included in the analysis and the distribution of the key health outcomes assessed: **(A)** crude test positivity, **(B)** crude case fatality, and **(C)** mortality risk. The figure also illustrates the geographical distribution of the six Hofstede cultural dimensions in these counties: **(D)** individualism (vs. collectivism), **(E)** uncertainty avoidance (vs. comfort with uncertainty), **(F)** long-term normative orientation (vs. short term), **(G)** power distance index (a greater level of hierarchy in society), **(H)** indulgence (vs. restraint), and **(I)** masculinity (vs. femininity) in each. The colored shading ranks each observation from high to low with the darker shading corresponding to greater value for each feature. For example, darker shaded observations in the **(D)** indicates greater degree of individualism vs. collectivism.

**Figure 2:**
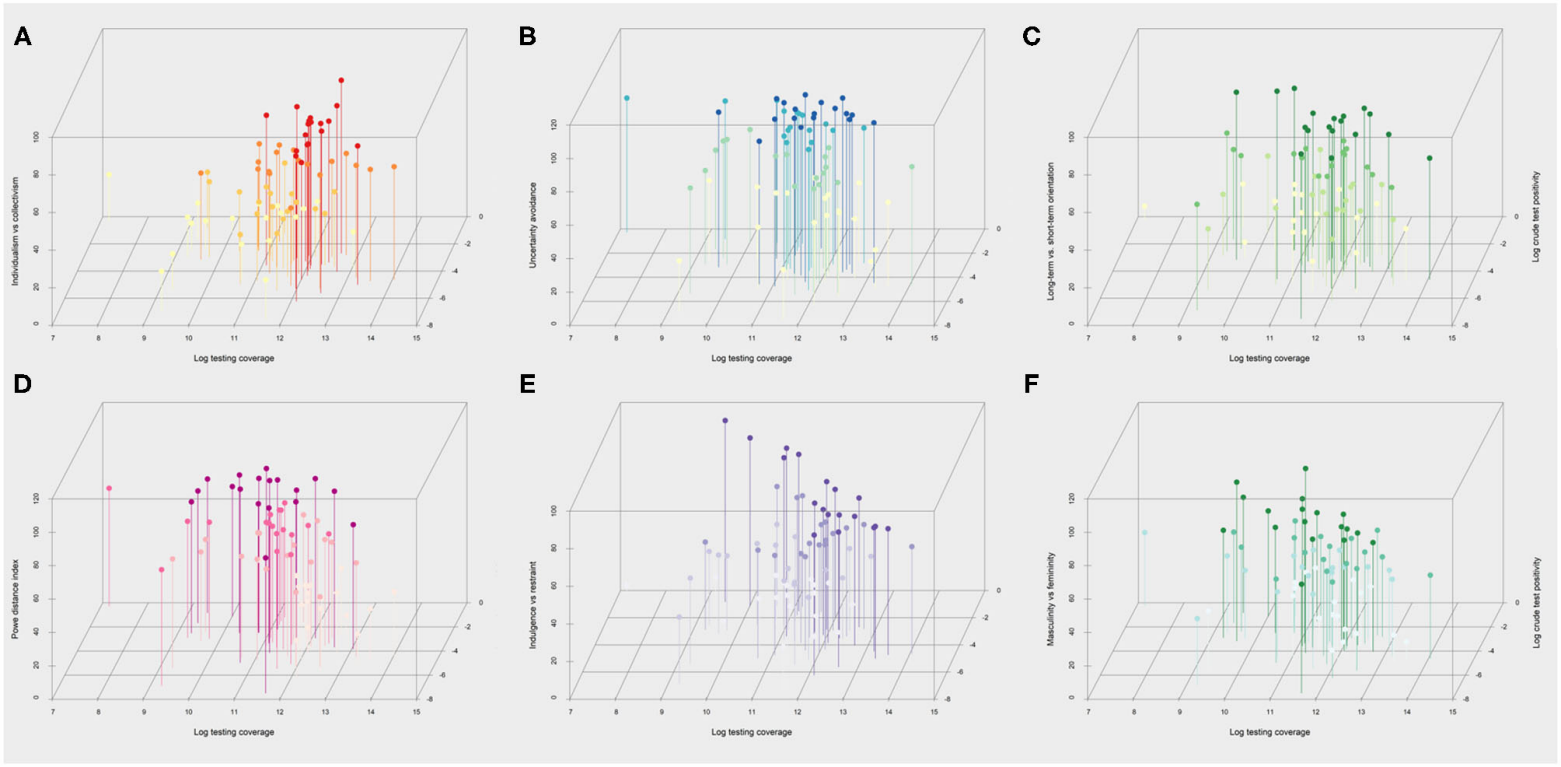
Scatterplots of the six cultural dimensions with a measure of infection spread and testing coverage over the first wave up to September 20, 2020. Scatterplots of Hofstede cultural dimensions (y-axis) vs. log testing coverage (x-axis) and crude test positivity (z-axis) across 73 countries included in the analysis. Higher values on the y-axis indicate a higher degree of **(A)** individualism (vs. collectivism), **(B)** uncertainty avoidance (vs. comfort with uncertainty), **(C)** tendency for long-term orientation (vs. short term), **(D)** power distance (a greater level of hierarchy), **(E)** indulgence (vs. restraint) **(F)** masculinity (vs. femininity) in society. The colored shading ranks each observation from high to low for each cultural dimension using four ordinal categories. For instance, darker shades in the **(A)** indicates greater level of individualism vs. collectivism.

**Figure 3:**
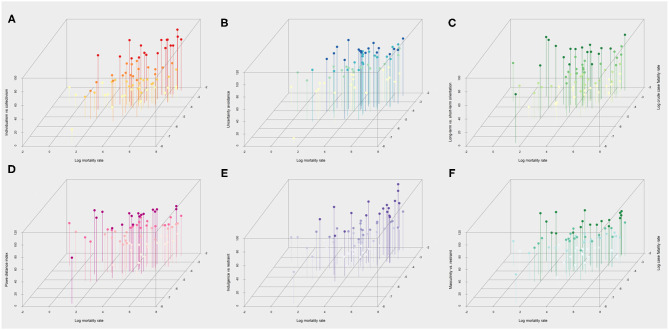
Scatterplots of the six cultural dimensions the two measures of fatality over the first wave up to September 20, 2020. Scatterplots of Hofstede cultural dimensions (y-axis) vs. log transformed mortality (x-axis) and case fatality risk (z-axis) across 73 countries included in the analysis. Higher values on the y-axis indicate a higher degree of **(A)** individualism (vs. collectivism), **(B)** uncertainty avoidance (vs. comfort with uncertainty), **(C)** tendency for long-term orientation (vs. short term), **(D)** power distance (a greater level of hierarchy), **(E)** indulgence (vs. restraint) **(F)** masculinity (vs. femininity) in society. The colored shading ranks each observation from high to low for each cultural dimension using four ordinal categories. For instance, darker shades in the **(A)** indicates greater level of individualism vs. collectivism.

The geographical distribution of the key COVID-19 related health outcomes (crude test positivity, crude case fatality, and mortality risk) in the 73 countries included in the analysis are illustrated in Figure 1, along with their respective cultural features. With respect to these cultural dimensions, Figure 2 depicts in more detail the relationship of each cultural dimension simultaneously with a measure of disease spread (i.e., crude test positivity) and testing coverage. The specific countries that correspond to each observation are presented in Supplementary Figure 1 (Panel A). The plots indicate a positive relationship between individualism (vs. collectivism) with testing coverage and a negative relationship with test positivity, while no discernible relationships are apparent with other cultural features. Similarly, Figure 3 depicts the relationship of each cultural dimension simultaneously with the two fatality outcomes (i.e., crude case fatality and mortality risk), the countries that correspond to these observations are presented in Supplementary Figure 1 (Panel B). This figure illustrates a noticeable positive correlation of individualism (vs. collectivism) with both fatality measures, as well as a similar, albeit weaker relationships of uncertainty avoidance and indulgence (vs. restraint) with both outcomes. Likewise, long-term (vs. short-term) normative orientation also displays a weak positive correlation, but only with mortality rate outcome.

### Pooled Estimates of Public Health Outcomes

**Figure 4:**
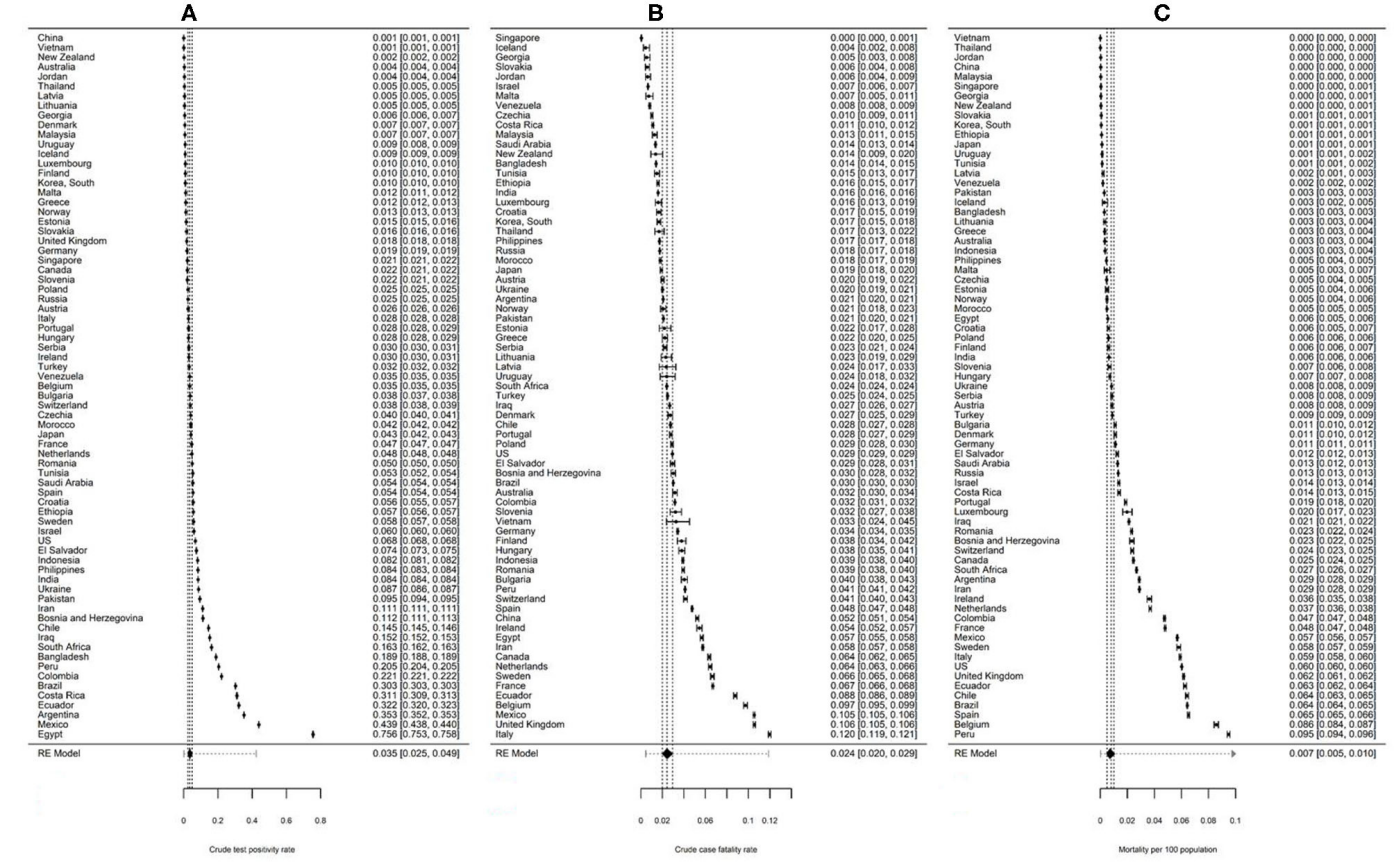
Forrest plot showing pooled public health outcomes over the first wave up to September 20, 2020. Figure showing forest plot of pooled **(A)** crude test positivity, **(B)** crude case fatality risk, and **(C)** mortality per 100 population attributable to COVID-19. A random-effects meta-analysis was used to pool estimates across countries using data from the final follow-up point (September 20, 2020). The values on the right represent the estimates for each country and their 95% confidence intervals. The position of the diamond indicates the value of the pooled random effects estimate for each outcome. The 95% confidence interval around each pooled estimate is indicated by the width of the diamonds and the prediction intervals are illustrated using the dotted lines.

The three outcomes for all 73 countries were pooled using random effects meta-analysis. The pooled estimates are depicted using Forrest plots in Figure 4. In brief, over the timeframe of the analysis, the pooled crude test positivity estimate was 3.5% (95%CI: 2.49–4.90, PI: 0.18–42.41) (Panel A), the pooled crude CFR was 2.4% (95%CI: 2.00–2.94, PI: 0.46–11.87) (Panel B), and the pooled COVID-19-attributed mortality risk was 85 deaths per million people (95%CI: 54.9–129.8, PI: 1.6–842.7) (Panel C). Of the three outcomes, test positivity appears to be the highest in Egypt; followed by several South American countries, nominally Mexico, Argentina, and Ecuador. In contrast East Asian and Pacific countries, including China, Vietnam, New Zealand, and Australia, displayed the lowest test positivity overall. With respect to case fatality estimates, individuals with identified infections in Italy, UK, and Mexico are the most likely to experience a fatal outcome; whereas infections identified in Singapore, Iceland, and Georgia were the least likely to experience fatalities. In terms of overall risk of COVID-19-attributed fatalities among the general population, Peru, and Belgium displayed the largest reported fatalities followed by Spain and several South American countries; whereas many East Asian countries such as Vietnam and Thailand appear to have the lowest mortality overall during this time frame.

### Model Specification

This variability in COVID-19 attributed outcomes during the first wave of the pandemic was further explored using random effects meta-regression analyses (Tables 2–**4**) by employing two different model specification approaches: a theory driven *a priori* model (Model 1) and an exploratory data-driven model developed using automated variable selection method (Model 2). In the *a priori* model (Model 1), we explored the effects of a predetermined set of predictors including underlying demographics, health system capacity, the epidemic timeline, and key cultural and political characteristics that may play a role in infectious disease dynamics, emergency preparedness and crisis management capacity of different settings. The exploratory model (Model 2) used a bootstrapping variable selection method in the model specification process to identify potentially relevant covariates from a larger set of predictors described in Table 1. We used these statistical approaches to specifically explore how collective cultural/behavioral differences could influence important public health outcomes.

**Table 2:** Random-effects meta-regression of crude test positivity risk over the first wave up to September 20, 2020.

	**MODEL 1: a priori model**				**MODEL 2: bootstrap variable selection**			
	**Crude test positivity risk**				**Crude test positivity risk**			
**Covariates**	**β**	**SE**	***P*-value**	**OR**	**β**	**SE**	***P*-value**	**OR**
Intercept	−1.7053	2.2885	–	–	−3.3940	2.1018	–	–
**Sociodemographic factors**								
GDP per capita ($1,000 USD, 2019)	0.0198	0.0144	0.175	1.02	–	–	–	–
Urban population (%)	–	–	–	–	−0.0233	0.0107	**0.034**	**0.98**
Population density (pop per km^2^)	0.0003	0.0002	0.130	1.00	0.0005	0.0002	**0.007**	**1.00**
Elderly dependency ratio (% of adults)	–	–	–	–	0.2968	0.1615	0.071	1.35
Proportion over 65 years (%)	−0.1332	0.0420	**0.002**	**0.88**	−0.6315	0.2581	**0.017**	**0.53**
Proportion overweight (%)	–	–	–	–	–	–	–	–
Proportion smoker (%)	–	–	–	–	–	–	–	–
**Pandemic–related factors**								
Time since 1st case (days)	−0.0116	0.0093	0.217	0.99	−0.0196	0.0086	**0.026**	**0.98**
Time since 100 cases (days)	–	–	–	–	0.0254	0.0075	**0.001**	**1.03**
Time since 1st death (days)	–	–	–	–	–	–	–	–
Testing coverage (*n*. tests per 10,000 pop)	−0.0318	0.0118	**0.009**	**0.97**	−0.0108	0.0085	0.212	0.99
**Health system strength**								
Healthcare workers (*n*. per 1,000 pop)	−0.0111	0.0571	0.847	0.99	–	–	–	–
Hospital beds (*n*. per 1,000 pop)	–	–	–	–	–	–	–	–
Health expenditure (% of GDP)	–	–	–	–	0.2145	0.0708	**0.004**	**1.24**
**Cultural characteristics**								
Individualism vs. collectivism	0.0063	0.0107	0.560	1.01	–	–	–	–
Uncertainty avoidance	0.0317	0.0098	**0.002**	**1.03**	0.0250	0.0077	**0.002**	**1.03**
Indulgence vs. restraint	–	–	–	–	–	–	–	–
Long–term vs. short–term orientation	–	–	–	–	–	–	–	–
Power distance	–	–	–	–	–	–	–	–
Masculinity vs. femininity	–	–	–	–	–	–	–	–
**Political characteristics**								
Polity (democracy vs. authoritarianism)	0.0418	0.0419	0.322	1.04	0.0544	0.0370	0.147	1.06
	pseudo-*R*^2^: 31%				pseudo *R*^2^: 46%			
	AIC:231.1 BIC:254.7				AIC:214.2 BIC:239.7			

*Random-effects meta-regression analysis of the crude test positivity risk at the last follow-up date in the main analysis (September 20, 2020) for 73 countries. Dependent variables were logit transformation to stabilize the variance of proportions. Random-effects meta-regression was used to explore the impact of cultural characteristics on fatalities while adjusting for important predefined covariates. The odds ratios (OR) represents the odds of a positive test upon exposure to a risk factor relative to no exposure. For example, an OR of 1.03 indicates that a one-unit increase uncertainty avoidance, we expect to see a 3% increase in the odds of a new positive test result across all test performed. Pseudo-R-squared value represent the proportion of heterogeneity explained by predictors included in the model. AIC, Akaike information criterion; BIC, Bayesian information criterion. Bold font indicates a statistically significant association with outcome at p < 0.05*.

### Culture as a Predictor of Infection Spread

After adjusting for potential confounders in the *a priori* model (Table 2, Model 1), we identified three covariates as statistically significant predictors of infection spread indicated by the crude test positivity metric. Indeed, of these covariates, population age had the largest impact on test positivity, whereby countries with a larger proportion of individuals over the age of 65 years displayed a significantly lower test positivity (OR:0.88), demonstrating that during this time period, having a younger population was associated with greater disease spread independent of other covariates. In relation to cultural characteristics, one cultural attribute, uncertainty avoidance, had the second largest significant effect on this outcome, such that societies with greater levels of discomfort with uncertainty experienced a small but statistically significantly increase in test positivity (OR:1.03) during this time. This result supports our theoretical expectation regarding uncertainty avoidance. However, individualism is not shown to impact infection spread in a significant manner. Furthermore, and unsurprisingly, we also found that countries with more liberal testing as implied by a greater testing coverage of the general population also had significantly lower test positivity (OR: 0.97) after adjusting for other covariates.

When we applied a data-driven bootstrap variable selection method to select relevant predictors from a larger set of potential covariates (Table 2, Model 2), three variables included in the original model were omitted (GDP per capita, healthcare workers per 1,000 population, and individualism). Instead, this model specification approach identified several other variables as potentially relevant predictors of infection spread including indicators of epidemic timing (i.e., time since 100 cases), health expenditure, urban population, and elderly dependency ratio.

Consistently with the theory-driven approach, in this model, uncertainty avoidance (OR:1.03) and older population age (OR:0.53) both displayed similar statistically significant associations with infection spread independent of other covariates. However, testing coverage no longer retained a statistically significant effect in this case. Moreover, unlike the *a priori* model, the bootstrap approach also identified a significant positive relationship between population density and test positivity. Similarly, a significant positive association was also apparent between the level of urbanization and test positivity independent of other predictors. Of these variables, health expenditure had the second largest effect on the test positivity following population age, whereby countries with a larger health expenditure as a proportion of GDP had a significantly higher test positivity (OR:1.24). In terms of the epidemic curve, we also found that countries with an earlier detection of the first case of SARS-CoV-2 had significantly lower disease spread (OR:0.98); whereas, those with greater time elapsed since the first 100 cases of infection to the time of the analysis exhibited a significantly greater infection spread (OR:1.03). In brief, the covariates included in Model 1 and Model 2 accounted for 31 and 46% of the total observed variability in this metric of disease spread with model fit statistics, suggesting that Model 2 is a slightly more parsimonious model relative to Model 1.

### Culture as a Predictor of Fatality Among Detected Infections

With respect to the crude case fatality outcome, after adjusting for potential confounders, five covariates were identified as statistically significant predictors of crude CFR in the *a priori* model (Table 3, Model 1). In terms of cultural attributes, we found that countries that demonstrate a tendency toward individualism, as opposed to collectivism on the Hofstede dimensions exhibited significantly higher crude CFR (OR:1.01). Similarly, we also found that societies that report a greater discomfort with uncertainty also displayed significantly higher crude CFR (OR:1.01). While these results highlight the presence of a small but significant effect of cultural attributes on case fatality and support our expectations, unsurprisingly non-cultural predictors had a greater level of impact on this outcome. For instance, settings with a better health system capacity as indicated by a larger number of hospital beds (OR:0.87), as well as nations with broader testing (OR:0.98) both demonstrated significant negative associations with this metric, with the former having the largest effect in terms of magnitude. Moreover, in relation to epidemic timing, we also found that countries with an earlier date of initial deaths on record displayed a small but significantly greater crude CFR (OR:1.02).

**Table 3:** Random-effects meta-regression of crude case fatality risk over the first wave up to September 20, 2020.

	**MODEL 1: a priori model**				**MODEL 2: bootstrap variable selection**			
	**Crude case fatality risk**				**Crude case fatality risk**			
**Covariates**	**β**	**SE**	***P*-value**	**OR**	**β**	**SE**	***P*-value**	**OR**
Intercept	−9.5118	1.3360	–	–	−10.6256	1.2843	–	–
**Sociodemographic factors**								
GDP per capita ($1,000 USD, 2019)	−0.0005	0.0084	0.950	1.00	–	–	–	–
Urban population (%)	–	–	–	–	−0.0297	0.0079	**<0.0001**	**0.97**
Elderly dependency ratio (% of adults)	–	–	–	–	0.1417	0.1004	0.164	1.15
Proportion over 65 years (%)	0.0256	0.0268	0.343	1.03	−0.2221	0.1629	0.178	0.80
Proportion overweight (%)	–	–	–	–	0.0326	0.0108	**0.004**	**1.03**
Proportion smoker (%)	–	–	–	–	−0.0201	0.0121	0.103	0.98
**Pandemic–related factors**								
Time since 1st case (days)	–	–	–	–	–	–	–	–
Time since 100 cases (days)	–	–	–	–	–	–	–	–
Time since 1st death (days)	0.0246	0.0066	**<0.0001**	**1.02**	0.0314	0.0060	**<0.0001**	**1.03**
Testing coverage (*n*. tests per 10,000 pop)	−0.0153	0.0070	**0.033**	**0.98**	−0.0112	0.0051	**0.033**	**0.99**
**Health system strength**								
Healthcare workers (*n*. per 1,000 pop)	0.0044	0.0330	0.895	1.00	–	–	–	–
Hospital beds (*n*. per 1,000 pop)	−0.1352	0.0514	**0.011**	**0.87**	−0.1055	0.0595	0.082	0.90
Health expenditure (% of GDP)	–	–	–	–	–	–	–	–
**Cultural characteristics**								
Individualism vs. collectivism	0.0147	0.0059	**0.015**	**1.01**	0.0123	0.0060	**0.047**	**1.01**
Uncertainty avoidance	0.0124	0.0052	**0.019**	**1.01**	0.0120	0.0056	**0.037**	**1.01**
Indulgence vs. restraint	–	–	–	–	0.0138	0.0055	**0.015**	**1.01**
Long-term vs. short-term orientation	–	–	–	–	0.0192	0.0063	**0.004**	**1.02**
Power distance	–	–	–	–	–	–	–	–
Masculinity vs. femininity	–	–	–	–	−0.0085	0.0044	0.057	0.99
**Political characteristics**								
Polity (democracy vs. authoritarianism)	0.0023	0.0226	0.920	1.00	–	–	–	–
	pseudo–*R*^2^: 29%				pseudo *R*^2^: 47%			
	AIC:161.9 BIC:185.5				AIC:143.8 BIC: 175.1			

*Random-effects meta-regression analysis of the crude case fatality risk at the last follow-up date in the main analysis (September 20, 2020) for 73 countries. Dependent variables were logit transformation to stabilize the variance of proportions. Random-effects meta-regression was used to explore the impact of cultural characteristics on fatalities while adjusting for important predefined covariates. The odds ratios (OR) represents the odds of a fatal outcome upon exposure to a risk factor relative to no exposure. For example, an OR of 1.03 indicates that a one unit increase the proportion of the population overweight, we expect to see a 3% increase in the odds of fatal outcome among infected individuals. Pseudo-R-squared value represent the proportion of heterogeneity explained by predictors included in the model. AIC, Akaike information criterion; BIC, Bayesian information criterion. Bold font indicates a statistically significant association with outcome at p < 0.05*.

When we applied a bootstrap variable selection method to select model variables from a larger set of potential covariates (Table 3, Model 2), the variables selected using the bootstrap method matched closely with the theory driven variables (Table 3, Model 1). However, the bootstrap method (Model 2) excluded three variables (GDP per capita, healthcare workers per 1,000 population and polity as relevant predictors) and instead included three additional cultural variables (indulgence vs. restraint, long-term vs. short-term orientation and masculinity vs. femininity) as pertinent covariates. In this model, all cultural dimensions except for masculinity vs. femininity displayed significant positive relationships with crude CFR (OR: 1.01–1.02). Although this was not statistically significant, masculinity vs. femininity still exhibited a negative relationship with this crude CFR estimate (OR: 0.99) that closely approached significance (*p* = 0.057).

In addition to cultural dimensions, the bootstrap method identified four additional variables as potentially relevant predictors: urban population, elderly dependency ratio, proportion of overweight adults, and proportion of smokers. Of these, only two were identified to have statistically significant associations with crude CFR: a higher degree of urbanization was associated with a lower crude CFR (OR:0.97), whereas having a greater proportion of overweight individuals was associated with a higher crude CFR (OR:1.03). In general, the covariates included in Model 1 and Model 2 accounted for 29 and 47% of the total observed variability in the crude CFR, respectively, with Model 2 indicating a more parsimonious model.

### Culture as a Predictor of Fatality Among the General Population

As with CFR, the overall mortality risk (Table 4) also demonstrated similar associations with selected predictors; however, with the theory-driven *a priori* model specification approach (Table 4, Model 1) only three covariates reached statistical significance, one of which was uncertainty avoidance (OR:1.05). In general, the covariate with the greatest impact on mortality risk was the number of hospital beds, which exhibited a statistically significant negative association with mortality (OR:0.73); while the covariate with the most modestly positive yet significant effect on this outcome was time since first death (OR:1.03). In contrast to the crude CFR metric, mortality risk did not display a statistical association with either testing coverage or with individualism.

**Table 4:** Random–effects meta-regression of crude mortality risk over the first wave up to September 20, 2020.

	**MODEL 1: a priori model**				**MODEL 2: bootstrap variable selection**			
	**Mortality risk (per 1,000 population)**				**Mortality risk (per 1,000 population)**			
**Covariates**	**β**	**SE**	***P*-value**	**OR**	**β**	**SE**	***P*-value**	**OR**
Intercept	−11.2412	2.6347	–	–	−12.3437	2.7791	–	–
**Sociodemographic factors**								
GDP per capita ($1,000 USD, 2019)	0.0112	0.0165	0.500	1.01	–	–	–	–
Urban population (%)	–	–	–	–	−0.0203	0.0134	0.135	0.98
Elderly dependency ratio (% of adults)	–	–	–	–	–	–	–	–
Proportion over 65 years (%)	−0.0269	0.0529	0.613	0.97	−0.0431	0.0481	0.375	0.96
Proportion overweight (%)	–	–	–	–	0.0531	0.0199	**0.010**	**1.05**
Proportion smoker (%)	–	–	–	–	−0.0368	0.0249	0.146	0.96
**Pandemic–related factors**								
Time since 1st case (days)	–	–	–	–	−0.0198	0.0136	0.152	0.98
Time since 100 cases (days)	–	–	–	–	0.0185	0.0095	0.055	1.02
Time since 1st death (days)	0.0261	0.0130	**0.050**	**1.03**	0.0326	0.0167	0.056	1.03
Testing coverage (*n*. tests per 10,000 pop)	−0.0039	0.0137	0.778	1.00	0.0087	0.0095	0.363	1.01
**Health system strength**								
Healthcare workers (*n*. per 1,000 pop)	0.0628	0.0648	0.337	1.06	–	–	–	–
Hospital beds (*n*. per 1,000 pop)	−0.3130	0.1017	**0.003**	**0.73**	−0.3382	0.1108	**0.003**	**0.71**
Health expenditure (% of GDP)	–	–	–	–	0.2371	0.0835	**0.006**	**1.27**
**Cultural characteristics**								
Individualism vs. collectivism	0.0193	0.0116	0.102	1.02	–	–	–	–
Uncertainty avoidance	0.0453	0.0101	**<0.0001**	**1.05**	0.0230	0.0094	**0.018**	**1.02**
Indulgence vs. restraint	–	–	–	–	–	–	–	–
Long–term vs. short–term orientation	–	–	–	–	0.0343	0.0120	**0.006**	**1.03**
Power distance	–	–	–	–	–	–	–	–
Masculinity vs. femininity	–	–	–	–	–	–	–	–
**Political characteristics**								
Polity (democracy vs. authoritarianism)	0.0541	0.0450	0.234	1.06	0.0696	0.0410	0.095	1.07
	pseudo–*R*^2^: 30%				pseudo *R*^2^: 47%			
	AIC:266.0 BIC: 289.6				AIC:252.6 BIC:280.0			

*Random-effects meta-regression analysis of the mortality risk at the last follow–up date in the main analysis (September 20, 2020) for 73 countries. Dependent variables were log transformed rates. Random-effects meta-regression was used to explore the impact of cultural characteristics on fatalities while adjusting for important predefined covariates. The odds ratios (OR) represents the odds of a fatal outcome upon exposure to a risk factor relative to no exposure. For example, an OR of 0.68 indicates that a one unit increase the number of hospital beds per 1,000 people, we expect to see a 32% decrease in the odds of mortality risk (per 1,000 people). Pseudo-R-squared value represent the proportion of heterogeneity explained by predictors included in the model. AIC, Akaike information criterion; BIC, Bayesian information criterion. Bold font indicates a statistically significant association with outcome at p < 0.05*.

When compared to the *a priori* model, variables selected using the statistical model specification approach (Table 4, Model 2) excluded three variables: GDP per capita, healthcare workers per 1,000 population and individualism vs. collectivism. Instead, this approach identified seven additional potentially relevant covariates: proportion of urban population, proportion of overweight, proportion of smokers, time since 1st case, time since 100 cases, health expenditure, and long-term vs. short-term normative orientation.

In total, five covariates in this model displayed statistically significant associations with mortality risk, two of which were cultural factors: uncertainty avoidance (OR:1.02) and long-term vs. short-term normative orientation (OR:1.03), both of which had a significant but moderately positive impact on mortality risk. As with the CFR, non-cultural predictors had a relatively larger impact on overall mortality. Of all the predictors, hospital beds per 1,000 population had the greatest impact on mortality risk (OR:0.61), displaying a statistically significant negative association with mortality as was the case with CFR. This was followed closely by health expenditure as a proportion of the GDP, which had the second largest impact on mortality (OR:1.27); displaying a significantly positive relationship with this outcome. Other predictors with a significant but more modest positive associations with this metric were the proportion of overweight (OR:1.05) and time elapsed since first death (1.03). With respect to the mortality risk, covariates included in Model 1 and Model 2 accounted for 28 and 47% of the total observed variability in mortality risk, respectively. In terms of model selection criteria Model 2 appeared to be the more parsimonious model, as was the case for crude CFR.

### Sensitivity Analysis

The impact of including potential outliers in the analyses was further explored through a sensitivity analysis. When compared to the main analysis, the removal of influential observations from the *a priori* model in the sensitivity analysis did not impact any of the findings for test positivity or mortality. However, upon removal of these observations, CFR no longer displayed a significant relationship with either cultural attribute (Supplementary Tables 1–3, Model 1). As for the data driven models, removal of outliers in this case also indicated generally robust findings with respect to cultural features; yet, there were some important differences in the cultural attributes identified specifically as predictors of CFR and test positivity.

More precisely, for test positivity, unlike the main analysis, the bootstrap selection approach identified a different set of cultural attributes as important predictors (Supplementary Table 1, Model 2). Following removal of influential observations, individualism (vs. collectivism) and long-term (vs. short-term) orientation were identified as relevant predictors of this metric instead of uncertainty avoidance; however, only individualism exhibited a statistically significant relationship (OR:0.98) in this analysis. Therefore, after removal of outliers, more collectivist societies (vs. individualist) appeared to display significantly higher test positivity after controlling for other factors in the model. Still, in the sensitivity analysis, the *a priori* model did not identify such a relationship (Supplementary Table 1, Model 1). In relation to non-cultural factors, the data driven model specification approach (Supplementary Table 1, Model 2) identified many of the same associations as the main analysis. However, in this case, elderly dependency ratio (OR:1.41) and overweight prevalence (OR:1.05) were additionally identified as having a statistically significant positive relationship with test positivity; whereas, testing coverage was associated with significantly lower test positivity (OR:0.98), which were not apparent in the main analysis.

In general, the crude case fatality outcome was the most sensitive to the removal of outliers from the analysis overall (Supplementary Table 2). For instance, following the removal of influential observations from the *a priori* model, the association of crude CFR with individualism and with uncertainty avoidance were lost (Supplementary Table 2, Model 1). Similarly, when key cultural characteristics were re-evaluated in the bootstrap model, crude CFR only retained a significant association with one cultural dimension: long-term vs. short-term orientation (OR:1.01); although indulgence vs. restraint was also selected as a potentially relevant predictor in this model, this was not statistically significant in the sensitivity analysis (Supplementary Table 2, Model 2). Additionally, however, the bootstrap model also identified a significant positive association between an older population age and crude case fatality (OR:1.07) as well.

As for the mortality risk outcome, the findings were robust for all the cultural dimensions which were identified in the initial analysis for both models (Supplementary Table 3, Model 1 and Model 2). Following the removal of influential observations, uncertainty avoidance retained a significant association with mortality in both models (OR:1.04). Similarly, long-term vs. short-term normative orientation also retained a significant association with mortality (OR:1.05) in the bootstrap model (Supplementary Table 4, Model 2). Moreover, in the sensitivity analysis, the bootstrap model additionally identified indulgence vs. restraint and power distance index as potentially relevant cultural predictors of mortality; with only indulgence vs. restraint (OR:1.04) exhibiting a statistically significant association with this metric.

### Extended Analysis

In a supplemental analysis we evaluated our models over a longer timeframe that covers the first two waves of the pandemic up to February 12, 2021. The results of this analysis are presented in Tables 5–7, Supplementary Figure 2. This extended analysis identified very similar findings to the main analysis, which focused on the first wave period. In this analysis, uncertainty avoidance was no longer associated with test positivity; however, this relationship was on the cusp of significance (*p* = 0.059). Power distance was also identified as a significant predictor of infection spread during this time in the data-driven model (OR:1.02). With respect to case fatality and mortality risk, both uncertainty avoidance (OR:1.01 and OR:1.04) and individualism (OR:1.01 and OR:1.03) retained a statistically significant associations with these outcomes, as did long-term vs. short-term orientation (OR:1.01 and OR: 1.02). Over this extended time frame, the data driven model also identified healthcare worker scarcity as being significantly associated with crude CFR (OR:0.96), which was not apparent in the analysis that focused on the first wave. Whereas, hospital bed capacity did not display any statistical association with any outcome over this time longer frame. Moreover, the polity index was also statistically related with a higher risk of mortality (OR:1.12) over the first two waves. In summary, cultural dimensions retained significant associations with outcomes even though this data cut is more likely to be impacted by both the emergence of variants-of-concern (VOC) in different parts of the world, the variable initiation of vaccinations focused on risk groups in higher-income settings, as well as other health system adaptations.

**Table 5:** Random-effects meta-regression analysis of the crude test positivity risk at the last follow-up date in the extended analysis (February 12, 2021) for 73 countries.

	**MODEL 1: a priori model**				**MODEL 2: bootstrap variable selection**			
	**Crude test positivity risk**				**Crude test positivity risk**			
**Covariates**	**β**	**SE**	***P*-value**	**OR**	**β**	**SE**	***P*-value**	***OR***
Intercept	6.1941	3.0688	–	–	4.3767	2.5753	–	–
**Sociodemographic factors**								
GDP per capita ($1,000 USD, 2019)	−0.0073	0.0118	0.537	0.99	–	–	–	–
Urban population (%)	–	–	–	–	−0.0164	0.0078	**0.040**	**0.98**
Population density (pop per km^2^)	0.0001	0.0002	0.584	1.00	–	–	–	–
Elderly dependency ratio (% of adults)	–	–	–	–	–	–	–	–
Proportion over 65 years (%)	−0.0097	0.0359	0.788	0.99	−0.1197	0.0720	0.102	0.89
Proportion over 80 years (%)	–	–	–	–	0.3764	0.2171	0.088	1.46
Proportion overweight (%)	–	–	–	–	–	–	–	–
**Pandemic–related factors**								
Time since 1st case (days)	−0.0277	0.0080	**0.001**	**0.97**	−0.0444	0.0063	**<0.0001**	**0.96**
Time since 100 cases (days)	–	–	–	–	0.0242	0.0062	**<0.0001**	**1.02**
Time since 1st death (days)	–	–	–	–	–	–	–	–
Testing coverage (*n*. tests per 10,000 pop)	−0.0047	0.0036	0.197	1.00	–	–	–	–
**Health system strength**								
Healthcare workers (*n*. per 1,000 pop)	−0.0126	0.0487	0.797	0.99	−0.0701	0.0335	**0.041**	**0.93**
Hospital beds (*n*. per 1,000 pop)	–	–	–	–	–	–	–	–
Health expenditure (% of GDP)	–	–	–	–	0.2165	0.0598	**0.001**	**1.24**
Out–of–pocket health expenditure (%)	–	–	–	–	–	–	–	–
**Cultural characteristics**								
Individualism vs. collectivism	0.0073	0.0091	0.427	1.01	–	–	–	–
Uncertainty avoidance	0.0158	0.0082	0.059	1.02	–	–	–	–
Indulgence vs. restraint	–	–	–	–	–	–	–	–
Long–term vs. short–term orientation	–	–	–	–	–	–	–	–
Power distance	–	–	–	–	0.0192	0.0063	**0.003**	**1.02**
Masculinity vs. femininity	–	–	–	–	–	–	–	–
**Political characteristics**								
Polity (democracy vs. authoritarianism)	0.0454	0.0353	0.203	1.05	0.0461	0.0296	0.125	1.05
	pseudo *R*^2^: 36%				pseudo *R*^2^: 53%			
	AIC:209.6 BIC:233.2				AIC:190.6 BIC:214.1			

*Dependent variables were logit transformation to stabilize the variance of proportions. Random-effects meta-regression was used to explore the impact of cultural characteristics on fatalities while adjusting for important predefined covariates. Pseudo-R-squared value represent the proportion of heterogeneity explained by predictors included in the model. OR, Odds ratio; AIC, Akaike information criterion; BIC, Bayesian information criterion. Bold font indicates a statistically significant association with outcome at p < 0.05*.

**Table 6:** Random-effects meta-regression analysis of the crude case fatality risk at the last follow-up date in the extended analysis (February 12, 2021) for 73 countries.

	**MODEL 1: a priori model**				**MODEL 2: bootstrap variable selection**			
	**Crude case fatality risk**				**Crude case fatality risk**			
**Covariates**	**β**	**SE**	***P*-value**	**OR**	**β**	**SE**	***P*-value**	**OR**
Intercept	−9.8586	1.9209	–	–	−10.2149	1.6963	–	–
**Sociodemographic factors**								
GDP per capita ($1,000 USD, 2019)	−0.0105	0.0068	0.128	0.99	–	–	–	–
Urban population (%)	–	–	–	–	−0.0204	0.0061	**0.002**	**0.98**
Population density (pop per km^2^)	–	–	–	–	−0.0002	0.0001	**0.007**	**1.00**
Elderly dependency ratio (% of adults)	–	–	–	–	0.0980	0.0754	0.199	1.10
Proportion over 65 years (%)	−0.0210	0.0231	0.367	0.98	−0.1731	0.1236	0.167	0.84
Proportion over 80 years (%)	–	–	–	–	–	–	–	–
Proportion overweight (%)	–	–	–	–	0.0283	0.0079	**0.001**	**1.03**
**Pandemic–related factors**								
Time since 1st case (days)	–	–	–	–	–	–	–	–
Time since 100 cases (days)	–	–	–	–	–	–	–	–
Time since 1st death (days)	0.0148	0.0056	**0.010**	**1.01**	0.0137	0.0047	**0.005**	**1.01**
Testing coverage (*n*. tests per 10,000 pop)	−0.0019	0.0021	0.363	1.00	–	–	–	–
**Health system strength**								
Healthcare workers (*n*. per 1,000 pop)	−0.0024	0.0280	0.932	1.00	−0.0399	0.0190	**<0.0001**	**0.96**
Hospital beds (*n*. per 1,000 pop)	−0.0167	0.0435	0.703	0.98	–	–	–	–
Health expenditure (% of GDP)	–	–	–	–	0.0965	0.0346	**0.007**	**1.10**
Out–of–pocket health expenditure (%)	–	–	–	–	0.0096	0.0051	0.065	1.01
**Cultural characteristics**								
Individualism vs. collectivism	0.0139	0.0050	**0.007**	**1.01**	0.0092	0.0042	**0.031**	**1.01**
Uncertainty avoidance	0.0132	0.0043	**0.003**	**1.01**	0.0055	0.0035	0.114	1.01
Indulgence vs. restraint	–	–	–	–	–	–	–	–
Long-term vs. short-term orientation	–	–	–	–	0.0126	0.0043	**0.005**	**1.01**
Power distance	–	–	–	–	–	–	–	–
Masculinity vs. femininity	–	–	–	–	–	–	–	–
**Political characteristics**								
Polity (democracy vs. authoritarianism)	0.0234	0.0192	0.228	1.02	–	–	**–**	**–**
	pseudo *R^2^*: 26%				pseudo *R^2^*: 52%			
	AIC:140.0 BIC:163.6				AIC:113.7 BIC: 143.0			

*Dependent variables were logit transformation to stabilize the variance of proportions. Random-effects meta-regression was used to explore the impact of cultural characteristics on fatalities while adjusting for important predefined covariates. Pseudo-R-squared value represent the proportion of heterogeneity explained by predictors included in the model. OR, Odds ratio; AIC, Akaike information criterion; BIC, Bayesian information criterion. Bold font indicates a statistically significant association with outcome at p < 0.05*.

**Table 7:** Random-effects meta-regression analysis of the crude mortality risk at the last follow-up date in the extended analysis (February 12, 2021) for 73 countries.

	**MODEL 1: a priori model**				**MODEL 2: bootstrap variable selection**			
	**Mortality risk (per 1,000 population)**				**Mortality risk (per 1,000 population)**			
**Covariates**	**β**	**SE**	***P*-value**	**OR**	**β**	**SE**	***P*-value**	**OR**
Intercept	−5.5394	4.3667	–	–	−4.8819	3.0459	–	–
**Sociodemographic factors**								
GDP per capita ($1,000 USD, 2019)	−0.0227	0.0155	0.147	0.98	−0.0215	0.0087	**0.016**	**0.98**
Urban population (%)	–	–	–	–	−0.0327	0.0095	**0.001**	**0.97**
Population density (pop per km^2^)								
Elderly dependency ratio (% of adults)	–	–	–	–	–	–	–	–
Proportion over 65 years (%)	−0.0078	0.0525	0.882	0.99	−0.1930	0.0747	**0.012**	**0.82**
Proportion over 80 years (%)	–	–	–	–	0.4775	0.2220	**0.036**	**1.61**
Proportion overweight (%)	–	–	–	–	0.0676	0.0140	**<0.0001**	**1.07**
**Pandemic–related factors**								
Time since 1st case (days)	–	–	–	–	−0.0323	0.0079	**<0.0001**	**0.97**
Time since 100 cases (days)	–	–	–	–	0.0324	0.0065	**<0.0001**	**1.03**
Time since 1st death (days)	−0.0006	0.0128	0.961	1.00	–	–	–	–
Testing coverage (n. tests per 10,000 pop)	0.0080	0.0048	0.100	1.01	0.0100	0.0032	**0.003**	**1.01**
**Health system strength**								
Healthcare workers (*n*. per 1,000 pop)	0.0523	0.0636	0.414	1.05	–	–	–	–
Hospital beds (*n*. per 1,000 pop)	−0.1164	0.0992	0.245	0.89	–	–	–	–
Health expenditure (% of GDP)	–	–	–	–	0.2644	0.0649	**<0.0001**	**1.30**
Out–of–pocket health expenditure (%)	–	–	–	–	–	–	–	–
**Cultural characteristics**								
Individualism vs. collectivism	0.0273	0.0114	**0.020**	**1.03**	–	–	–	–
Uncertainty avoidance	0.0421	0.0098	**<0.0001**	**1.04**	–	–	–	–
Indulgence vs. restraint	–	–	–	–	–	–	–	–
Long–term vs. short–term orientation	–	–	–	–	0.0186	0.0073	**0.014**	**1.02**
Power distance	–	–	–	–	0.0113	0.0075	0.139	1.01
Masculinity vs. femininity	–	–	–	–	–	–	–	–
**Political characteristics**								
Polity (democracy vs. authoritarianism)	0.1022	0.0437	**0.023**	**1.11**	0.1122	0.0307	**0.001**	**1.12**
	pseudo *R*^2^: 41%				pseudo *R*^2^: 74%			
	AIC:246.6 BIC: 270.6				AIC:195 BIC:224			

*Dependent variables were log transformed rates. Random-effects meta-regression was used to explore the impact of cultural characteristics on fatalities while adjusting for important predefined covariates. Pseudo-R-squared value represent the proportion of heterogeneity explained by predictors included in the model. OR, Odds ratio; AIC, Akaike information criterion; BIC, Bayesian information criterion. Bold font indicates a statistically significant association with outcome at p < 0.05*.

## Discussion

It has been suggested that cultural factors can define the pre-existing (or, baseline) social and behavioral characteristics of societies and help to modulate both the public policy response and individuals' behavioral responses to the crisis in ways that theoretically impact infection transmission dynamics and fatalities (Bavel et al., 2020; Dheer et al., 2020; Ruhi, 2020; West et al., 2020). Indeed, numerous studies have shown that cultural factors can influence infectious disease dynamics, vaccination rates, infection prevention and control practices, and related health outcomes (Fincher et al., 2008; Borg, 2014a,b; Betsch et al., 2017). For instance, cultural attributes have been shown to predict almost half of the variance in Methicillin-resistant *Staphylococcus aureus* infections among European countries (Borg, 2014a). However, the impact of cultural/behavioral attributes in the context of the COVID-19 pandemic has generally been overlooked. To address this gap, we used meta-analytic methods to explore the extent to which the six independent cultural characteristic of nations, as described by Hofstede, can explain the global variability of COVID-19 attributed public health outcomes during the first wave of the pandemic, focusing on three related outcomes: test positivity (as a proxy for disease spread), case fatality risk, and mortality risk.

The main analyses focused exclusively on the first wave since the societal reactions to the initial wave of the pandemic are more likely to represent an immediate reaction to an acute crisis situation. Therefore, outcomes during this time are perhaps more likely to be directly driven by socio-cultural factors that represent the baseline behaviors as well as the immediate behavioral shifts or reactions to such a situation in contrast to economic concerns, which likely play a relatively greater role in shaping the responses and outcomes during the subsequent and more prolonged stages of the pandemic. Indeed, previous work has shown that cultural attributes can account for the variability in reactions to acute social crises (Kayser et al., 2008). Moreover, current evidence strongly suggests that the two initial waves of the epidemic have largely different characteristics in terms of the sociodemographic characteristics of individuals who have acquired the infection (Seligmann et al., 2020); consequently, the extent to which cultural factors can impact outcomes during a more prolonged crisis remain to be assessed.

In summary, the findings of this analysis highlight that certain country-level cultural/behavioral distinctions play a small but significant role in accounting for the severity of the COVID-19 crisis, independent of other important confounders (i.e., population age, economic capacity, health system strength, etc.,). Concerning, test positivity, we identified uncertainty avoidance as a significant predictor in the main analysis, which was robust following the removal of influential observations in the theory driven modeling approach. With respect to the two fatality outcomes, long-term normative orientation (vs. short term) generated the largest and the most consistent impact on fatalities, followed by uncertainty avoidance.

More specifically, in relation to the long-term (vs. short-term) orientation dimension, we found that a one-unit increase in a society's preference for long-term normative orientation results in a statistically significant ~1–2% increase in the odds of fatalities among infected cases and a 5% increase in the odds of a COVID-19-attributed mortality in the general population. This indicates that societies with a cultural orientation that prioritizes short-term phenomenon taking place were better able to mitigate fatalities during this timeframe, albeit this may be at the expense of more downstream or long-term outcomes. It may be that a greater emphasis on short-term, or immediate, events may prove to be somewhat beneficial when dealing with acute crisis situations. Typically, East Asian and European countries tend toward long-term orientation, whereas African, Islamic, South American, and Anglo-American countries tend toward short-term orientation (Hofstede and Minkov, 2010; Hofstede et al., 2010). In general, societies with long-term normative orientation tend to be more adaptive, less ideological, and future-focused, whereas those with short-term orientation tend to focus on past and present, respect tradition, norms and social obligations (Hofstede and Minkov, 2010; Hofstede, 2011). In countries with a preference for short-term orientation, a greater focus on the present may lead to stricter emergency measures, quicker reactions to a crisis, or a better compliance with procedures that focus more specifically on immediate difficulties.

Similarly, we also found that a cultural tendency toward uncertainty avoidance (i.e., greater discomfort with and resistance to unfamiliar phenomena) was also associated with higher fatalities for both outcomes: a one-unit increase in uncertainty avoidance was associated with a ~1% increase in the odds of a fatal outcome among infected cases and a ~3–5% increase in mortality risk, which was robust to the removal of outliers only for the mortality outcome. Similarly, we also found that a unit increase in uncertainty avoidance was also associated with a ~3% increase in the odds of a positive test result, suggesting that this cultural attribute may also influence the infection dynamics. Hofstede describes this dimension as a measure of a country's ability to adapt and cope with ambiguity (Hofstede, 2011). This indicates the degree of discomfort with unstructured, unknown and unexpected situations (Hofstede, 2011; Borg, 2014a). Societies with high uncertainty avoidance tend to be more resistant to change and therefore, paradoxically, more risk-tolerant (Borg, 2014a). Typically, this characteristics is more common in countries with a high degree of bureaucracy (Borg, 2014a). For instance, Southern and Eastern European countries display greater uncertainty avoidance, whereas Northern European countries tend to rank lower in this attribute (Hofstede, 2011). Past research has highlighted a negative relationship between uncertainty avoidance with both prosocial behavior (e.g., volunteerism) and rapport building with patients (Meeuwesen et al., 2009; Smith, 2015; Stojcic et al., 2016). Taken together, higher degrees of uncertainty avoidance could lead to weaker social responses, ineffective communication strategies and less attention given to vulnerable groups; three factors that can worsen such a crisis.

Additionally, we found that a one-unit increase in individualism (vs. collectivism) resulted in a ~1% increase in the odds of a fatal outcome among infected individuals. This suggests that individuals who became infected in more individualist societies may be those that belong to more socially vulnerable subgroups (i.e., elderly populations in long-term care); and may signify a greater reliance of institutional support for such populations where outbreaks may have had excessively negative effects on case fatality. In line with this observation, previous research has also demonstrated that individualist families tend to rely more on formal support in regards to eldercare in comparison to collectivists ones where the family is the primary caregiver (Pyke and Bengtson, 1996). However, in the current analysis, this statistical association was lost following the removal of potential influential observations from the analysis. Moreover, in the context of test positivity, upon removal of outliers, in the data driven model, we also found that a unit increase in individualism (vs. collectivity) resulted in 2% lower odds of having a positive test; though this effect was not apparent in the main analysis. While, the impact of this cultural dimension on public health outcomes appears to be less consistent, there exist theoretical reasons to expect some relationship between this dimension and the outcomes assessed. With respect to this dimension, in general, many European and Anglo-American countries tend strongly toward individualism, whereas Asian countries display more collectivist attitudes (Kitayama et al., 2009; Triandis, 2018). Individualism has often been equated with neo-liberal socioeconomic policies that tend to undermine social welfare and lead to weak collective protections (Marshall and Peters, 2002). As well, individualist attitudes may more broadly lead to social behavior that focuses on the individual rather than the collective well-being. For instance, in previous investigations, collectivist societies have been shown to be more effective in reducing the transmission of pathogens during outbreaks vs. individualistic ones (Fincher et al., 2008; Morand and Walther, 2018). Likewise, individuals from more individualistic countries on the Hofstede dimensions have also been shown to have lower vaccination intentions (Betsch et al., 2017). However, in these context a communication of the concept of herd immunity was shown to be able to improve vaccination intentions particularly in societies that lack a collectivistic baseline stance (Betsch et al., 2017). Nevertheless, the interaction between cultural and behavioral phenomena is complex: studies have also suggested that collectivism may have developed as a more prominent cultural feature in regions that have historically had a higher burden of pathogens; as certain behavioral manifestations of collectivism have been theorized to hamper pathogen transmission (Fincher et al., 2008).

Moreover, findings also reveal some association between indulgence vs. restraint with fatality outcomes, although this is much less consistent than other cultural dimensions. In the current analysis, having a more indulgent (vs. restraint) society resulted in a 1% increase in the odds of a fatal outcome for infected cases; though this statistical relationship was only apparent in the data-driven model and was not robust to the removal of potential outliers. Similarly, a 4% increase in the odds of mortality risk level in the general population was also detected per unit increase in indulgence upon removal of outliers in the bootstrap model. Typically, indulgent societies are more extraverted and place a greater emphasis on leisure, whereas restraint societies tend to be regulated by strict social norms, and more inclined to have a fatalistic outlook. Generally, many South and North American countries, and certain North European countries (e.g., Sweden, The Netherlands) tend toward indulgence; whereas some Islamic countries (e.g., Pakistan, Egypt) and Eastern European countries (e.g., Russia, Ukraine) tend toward restraint.

Likewise, masculinity (vs. femininity) also did not display any substantial effect on outcomes. This characteristic refers to social gender roles. In masculine societies, emotional gender roles are described to be more distinct; whereas in feminine societies such a role separation is less apparent. Characteristically, assertiveness, and heroism tend to be more admired in masculine societies, while sympathy for more vulnerable groups are more typical in feminine cultures. On average, many North and South American, Central European, and East Asian countries tend toward masculinity, while certain North European countries tend strongly toward a feminine outlook (e.g., Sweden and Norway) (Hofstede et al., 1998).

Finally, we also did not identify any significant association between the power distance index and any health outcomes in the analysis. Although the extended analysis covering the first 2-waves identified this as a predictor of test positivity. This index measures the level of hierarchy within a society and is an indicator of the extent of deference given by less powerful members in society toward authority figures (e.g., governmental officers) (Hofstede et al., 2010; Hofstede, 2011). Moreover, societies that rank higher on the power distance index also tend to have more centralized decision-making, lower accountability, as well as a larger degree of income inequity (Hofstede, 2011). High power distance societies tend to therefore have less inclusive and participative decision-making and more bureaucratic procedures (Khatri, 2009). Typically, Eastern European and Asian countries rank higher on the power distance index, while Western European and North American countries rank lower (Hofstede et al., 2010). In societies with a lower degree of power distance, a decentralization of power may theoretically enable more efficient and more locally focused decision-making during a crisis. Though, we found no statistical association of this dimension with any outcomes assessed.

Indeed, non-cultural factors played a greater role in explaining much of the global variability in fatalities expected. For test positivity, testing coverage had the strongest impact, whereby a unit increase in testing coverage led to a 12–53% reduction in crude test positivity, depending on the model. In relation to case fatalities, we found that health system resources constraints had the largest impact on case fatalities. A one unit increase in the number of hospital beds per 1,000 individuals led to a ~13–15% reduction in the odds of a fatal outcome among infected cases and a 32–41% reduction in mortality risk. We also found that with each day elapsed since the first death on record there was a modest increase in case fatalities of 2–3% and in mortality risk of 3–6%. For these outcomes, a one unit increase in testing coverage was also associated with a statistically significant 2–3% reduction in fatalities among infected cases, indicating that countries with increased health system capacity in terms of testing coverage likely identify more asymptomatic cases resulting in lower estimates of crude case fatality. However, we also find that testing coverage is similarly associated with a statistically significant but more modest 1% reduction in the odds of mortality, although this is only significant in the bootstrap model and is sensitive to the removal of outliers. Finally, the findings also highlight the important role of certain comorbid conditions. For instance, a one unit increase in the proportion of overweight individuals results in a 3–7% increase in fatalities among infected cases and a 6–8% increase in the overall mortality risk. Finally, a one percent increase in the proportion of the population aged over 65 years also results in a 12–51% increase in test positivity as well as a 7% increase in the odds of mortality for infected cases; yet, the latter result is only apparent following the removal of outliers from the analysis. These findings indicate that, after having controlled for other important national characteristics, countries with an older demographic composition typically experience a lower disease spread but have greater fatalities.

In summary, while not all cultural dimensions display a relationship with the public health outcomes of the pandemic, the current analysis consistently found statistical associations between uncertainty avoidance and long-term normative orientation. Furthermore, individualism (vs. collectivism) and indulgence (vs. restraint) are also shown to impact some of the COVID-19 health outcomes. These findings therefore support our expectations in relation to uncertainty avoidance and individualism, but underscore that other cultural attributes also matter. Ultimately, the results support the assertion that cultural factors can modulate such outcomes after having controlled for important confounders. Further, in these analyses, cultural factors, together with demographic, economic, and health system characteristics together could explain ~31–46% of the variability in test positivity, 29–47% of the variability in case fatalities and 28–44% in mortality risk during the initial wave of the pandemic. The results suggest that in such public health crises, baseline cultural factors may play some role in influencing key outcomes (Betsch et al., 2017).

While we focus above on cultural and institutional motives to explain the link between cultural constructs and COVID-19 outcomes, individual-level behavioral phenomena should not be ignored. Indeed, the Hofstede model of cultural constructs has been linked to a variety of collective behaviors (see, for example, Luthar and Luthar, 2002; Manrai et al., 2011). In the COVID-19 crisis, as with any major health crisis, both collective and individual behaviors are important considerations that need to be taken when account in planning effective response strategies (Chen et al., 2017). While it is difficult to pinpoint the specific individual-level mechanisms that underlie our results, the growing literature on COVID-19 provides some insight. For instance, our finding that relates long-term oriented cultures with greater fatalities is consistent with results from Wang (2021), which suggest that long-term orientation leads to lesser social distancing. In the case of individualism, research has demonstrated that individualist countries implement less stringent measures to combat COVID-19 (Rapson, 2021); therefore, undoubtedly leading to less behavioral modifications aimed at curbing the epidemic. Furthermore, according to Bazzi et al. (2021), individualism can undermine prosocial behavior as it is linked to lesser mask usage and social distancing practices. As for indulgence, the hedonistic nature of indulgent cultures might hinder authorities ability to have their citizens respect measures aimed at curbing the COVID-19 crisis (Messner, 2020). Lastly, the fact that uncertainty avoidant cultures are linked with more inefficient governance practices and with leadership styles that hinder individual- and team-level innovation (Borg, 2014a; Laukkanen, 2015; Watts et al., 2020) surely contributes to ineffective decision-making during a crisis. Moreover, uncertainty avoidance has also been shown to be associated with the belief of COVID-19 conspiracy theories (Alper et al., 2020); potentially leading to a greater wariness of new public policies. However, for a more comprehensive understanding of the mechanisms that connect cultural attributes with individual behavior in the COVID-19 crisis, further research is necessary.

Further, growing research is now starting to focus more prominently on the role of individuals' personality traits in explaining compliance with COVID-19 measures (Blagov, 2020). Specifically, neuroticism has been shown to lead individuals to be more concerned about the crisis, whereas conscientiousness leads individuals to take more precautions (Aschwanden et al., 2021). Moreover, empathy was also found to be an important factor in determining adherence to measures aimed at curbing the epidemic (Pfattheicher et al., 2020; Zirenko et al., 2021); while fear has been found to modifying behavior toward COVID-19 measures (Harper et al., 2020). Furthermore, even the personality of key decision-makers has been shown to have a significant impact on governmental responses to the pandemic (Medeiros et al., 2021). Overall, these individual-level characteristics likely have an impact on important public health outcomes of the pandemic. Nevertheless, while we agree with Zirenko et al. (2021) that cultural contexts surely mediate the impact of individual personality traits on responses to COVID-19 measures, there is a need for further research into the interaction between social contexts and individual characteristics before the connection joining culture and individual behavior can be better understood.

Taken together, this study makes important contributions to the current scholarship by (1) examining data from the initial phase of the pandemic, where cultural attributes may shape baseline behavioral responses to such a crisis; (2) focusing on a collection of countries with measured cultural dimensions and which represent an overwhelming majority (~93%) of reported infections worldwide; (3) exploring the variability in a range of relevant public health outcomes across countries taking into account important demographic, social, economic, and cultural factors; (4) additionally adjusting for domestic political factors in relation to governance and transparency, which may directly or indirectly influence outcomes, (5) evaluating the robustness of findings, and (6) lastly, being the first study, to our knowledge, to demonstrate the extent to which cultural attributes can impact these important outcomes.

However, the study also has limitations. The first limitation pertains to the accuracy of the estimated outcomes. The purpose of this study is not to generate a precise global estimate of the infections, CFR or mortality rate, which has been previously attempted by others using a variety of statistical approaches (Basu, 2020; Ruan, 2020; Verity et al., 2020; Wu et al., 2020). Rather, the intent is to explore the observed variation of estimates of these outcomes as collected and reported by governments in response to the pandemic. Therefore, we only estimate the crude test positivity, crude CFR and mortality. With respect to the crude CFR metric, it is important to note that the denominator here includes unresolved (or active) cases resulting in a time-lag bias that likely underestimates the true CFR, particularly in the earlier instances of the outbreak. Nonetheless, the estimated crude CFRs in this study are more likely to be an overestimate owing to the relatively greater influence of ascertainment bias (i.e., the under-detection of mild and asymptomatic cases resulting from undertesting). Indeed, we find that crude CFR is significantly lower with greater testing coverage of the population, suggesting that expanded testing should reduce CFR estimates by identifying more mild infections. Further, a higher testing coverage could also reflect a better capacity for contact tracing and isolation, which may reduce onward transmission particularly among high-risk groups. A second limitation is related to residual variability resulting from the inconsistency in recording COVID-19-attributable deaths across nations. Additionally, we have also not evaluated the potential impact of divergent medical management practices; however, as no known effective treatment or vaccine for SARS-CoV-2 was available during the first wave of the pandemic, demographic factors, comorbidities and health system resource capacity along with the behavioral responsiveness of societies (both governmental and individuals) are more plausible explanations for the variability in such outcomes for that timeframe. Moreover, another limitation, particularly pertinent to the data-driven models is the risk of false positive findings. An added caveat of such ecological approaches is related to aggregation bias, whereby associations identified in a population-level analysis may not always reflect similar relationships at the individual level. Finally, the study tends to omit many African nations due to data availability making it difficult to generalize findings to these settings.

In addition to these issues, the use of the Hofstede model in the current study also merits some discussion. While the Hofstede model of cultural dimensions is a widely accepted and used tool, it has also been the target of criticism (see McSweeney et al., 2016 for detailed critiques of Hofstede's model, as well as Williamson, 2002; Taras and Steel, 2009 for detailed discussions on those criticisms). For instance, Hofstede (2001) argues that national cultures are rather stable and tend to change very slowly, taking as long as a century to accrue substantial changes (Hofstede, 2001). Yet, research has demonstrated that cultural values held by individuals are subject to change much more rapidly (Inglehart and Baker, 2000; Inglehart and Welzel, 2001). Hofstede (2002) addresses such critiques by highlighting the (very) long-term nature of culture's roots as well as the stability of his cultural dimensions through several longitudinal surveys (Hofstede, 2002). Another important critique of Hofstede's dimensions is related to the implied uniformity of national culture. Indeed, others have shown that cultural values vary within a country along regions and/or social groups (Au, 1999; Conway et al., 2001); casting some shadow on the accuracy of a uniform “national culture” (Bock, 1999). However, Hofstede (2001) argues that national institutions (e.g., political institutions) have a significant influence on the values that constitute national culture.

Further, cultural differences among regional and social groups have not been shown to undermine the overall homogeneity of national cultures (Mazanec et al., 2015). There is also a pragmatic aspect for focusing on national culture. Hofstede (2002) argues that while the country-level may not be very granular, it is generally an appropriate analytical unit that allows for adequate global comparison. In terms of our own study, a sub-national level granularity is impractical, even in advanced democracies were data at this level may not be attainable. A final point is related to the influence that cultural dimensions, as described by Hofstede, are assumed to have on individuals. The arrows of causation between determinism (i.e., culture being the cause of national- and individual-level outcomes) and voluntarism (i.e., the influence of individual free-will) might not always be clear (Erez and Gati, 2004; McSweeney et al., 2016). However, this deterministic aspect, which does not solely rely on complete individual agency, is also seen as one of the strengths of Hofstede's model (Venkateswaran and Ojha, 2019; Venkateswaran and George, 2020).

## Conclusions

In conclusion, this analysis suggests that an assessment of underlying cultural/behavioral and demographic characteristics along with health system constraints should contribute to better-suited and more effective public health and emergency preparedness measures. Specifically, our findings highlight that a society's cultural and behavioral attributes are also important factors that can independently impart a small but significant influence on key public health outcomes during such a crisis. As a result, policies devised during similar situations should consider the cultural context of societies and should bear in mind these differences when evaluating the transferability and implementation of divergent and seemingly successful policy approaches from one context to another. Moreover, as the pandemic evolves into a more chronic crisis and takes on a more long-term direction, the direct influence of cultural attributes may vary; though, as of February 2021, there is no indication of such an attenuation of culture's impact on COVID-19 related health outcomes. Nevertheless, future research should compare the impact of cultural attributes on long-term outcomes of the pandemic in ways that cover both health and economic dimensions of the crisis.

## Data Availability Statement

Publicly available datasets were analyzed in this study. This data can be found at: https://osf.io/6sezm/?view_only=f5b69daaffa44707af434305021f384f.

## Author Contributions

AE collected and analyzed the data and drafted the manuscript. MM contributed to the analysis, interpretation of the results, and the drafting of the manuscript. All authors contributed in the revision of the manuscript, gave final approval for the version to be published manuscript, had full access to all the data during the study, and had final responsibility for the decision to submit for publication.

## Conflict of Interest

The authors declare that the research was conducted in the absence of any commercial or financial relationships that could be construed as a potential conflict of interest.

## Footnotes

^1^For countries where there were available data for at least four out of the six dimensions in the original Hofstede data, estimates of missing dimensions have been estimated through subsequent research (available though https://www.hofstede-insights.com/product/compare-countries). To cover countries with important epidemics and broader geographical coverage, we also included data from Egypt, Ethiopia, Iraq, Jordan, Saudi Arabia, Bosnia and Herzegovina, Georgia, Ukraine, and Iceland. While, direct measurements of most cultural dimensions were not available for these countries, the full complement of dimensions had been estimated through subsequent research beyond the original dataset.
