# Supplementary figures and images for: Exploring the Effect of Collective Cultural Attributes on Covid-19-Related Public Health Outcomes
Source: Front Psychol. 2021 Mar 23;12:627669. doi: 10.3389/fpsyg.2021.627669 (PMC8021731; doi:10.3389/fpsyg.2021.627669)

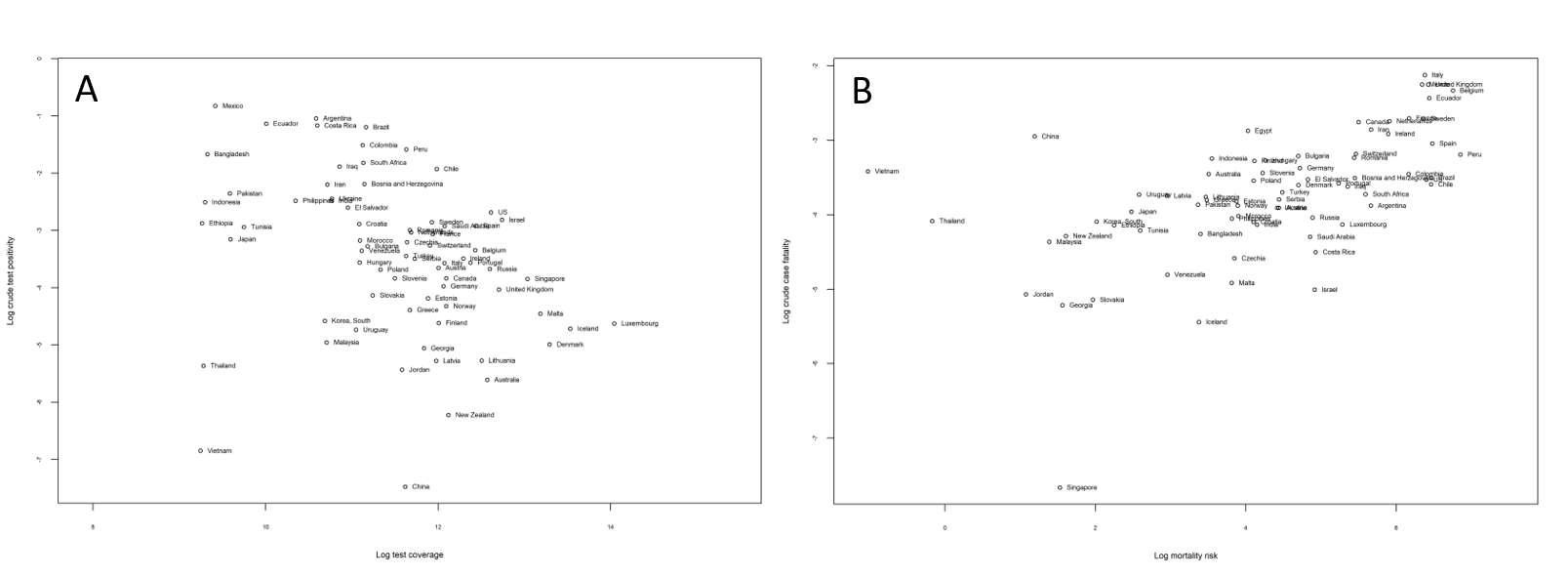

Supplement: Supplementary file 1 [file Image_1.tif]

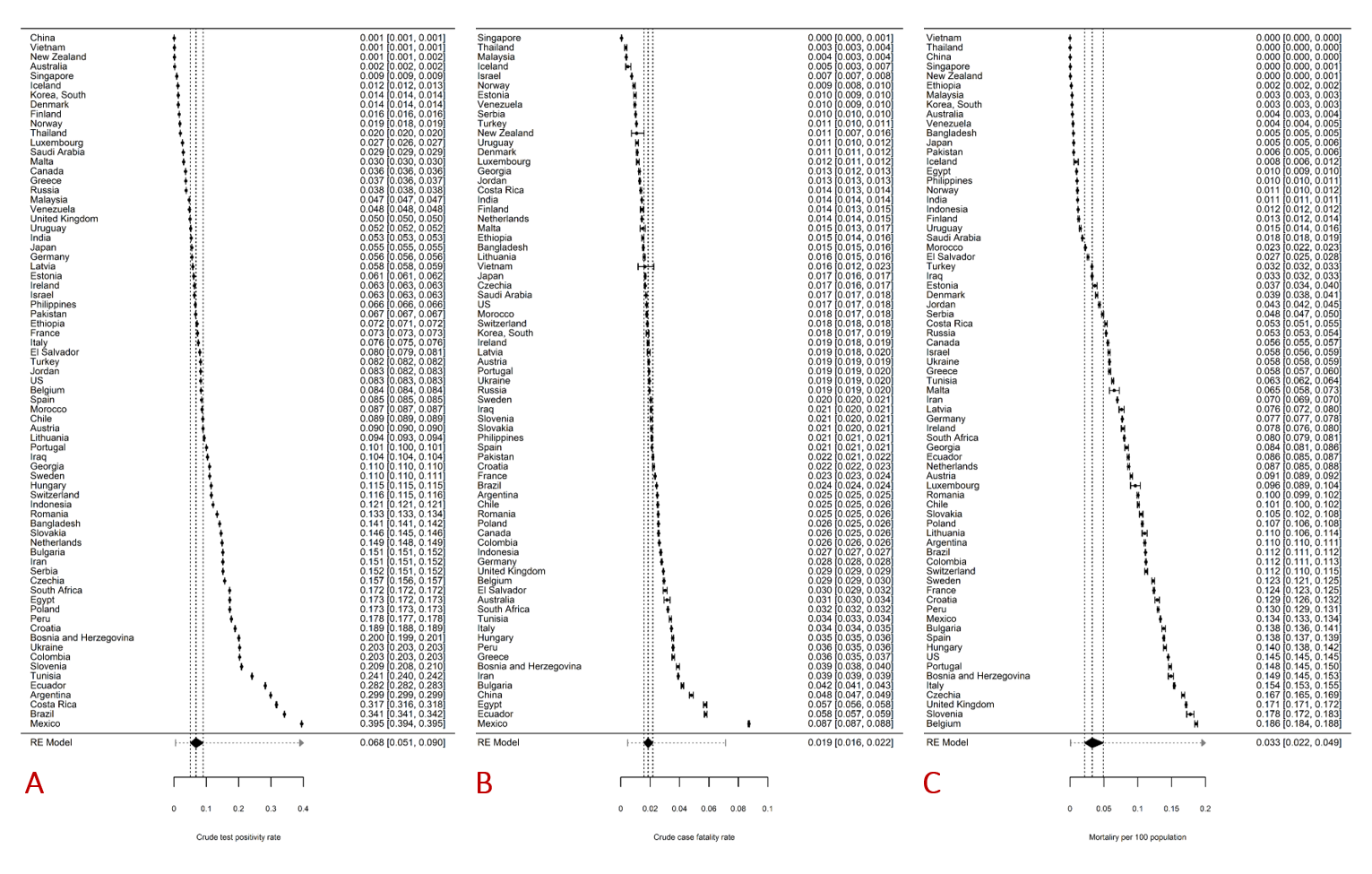

Supplement: Supplementary file 2 [file Image_2.tif]
