# Supplementary material for: Exploring the Effect of Collective Cultural Attributes on Covid-19-Related Public Health Outcomes
Source: Front Psychol. 2021 Mar 23;12:627669. doi: 10.3389/fpsyg.2021.627669 (PMC8021731; doi:10.3389/fpsyg.2021.627669)
Supplement: Supplementary file 3 [file Table_1.docx]

### **Supplemental Table 1: Sensitivity analysis showing the impact of outliers on predictors of test positivity risk**

| ***EXCLUDING CHINA, EGYPT, SINGAPORE  LUXEMBOURG, AND JORDAN*** | **MODEL 1: a priori model** | | | | **MODEL 2: bootstrap variable selection** | | | |
| --- | --- | --- | --- | --- | --- | --- | --- | --- |
|  | **Crude test positivity risk** | | | | **Crude test positivity risk** | | | |
| **Covariates** | **β** | **SE** | **P-value** | **OR** | **β** | **SE** | **P-value** | **OR** |
| *Intercept* | *-2.0226* | *2.1658* | *-* | *-* | *-8.3890* | *2.3805* | *-* | *-* |
| ***Sociodemographic factors*** |  |  |  |  |  |  |  |  |
| GDP per capita ($1,000 USD, 2019) | 0.0111 | 0.0144 | *0.443* | *1.01* | - | - | *-* | *-* |
| Population density (pop per km2) | - | - | *-* | *-* | 0.0013 | 0.0005 | ***0.019*** | ***1.00*** |
| Urban population (%) | - | - | *-* | *-* | -0.0191 | 0.0109 | *0.087* | *0.98* |
| Elderly dependency ratio (% of adults) | - | - | *-* | *-* | 0.3462 | 0.1466 | ***0.022*** | ***1.41*** |
| Proportion over 65 years (%) | -0.1352 | 0.0391 | ***0.001*** | ***0.87*** | -0.7053 | 0.2394 | ***0.005*** | ***0.49*** |
| Proportion overweight (%) | - | - | *-* | *-* | 0.0446 | 0.0169 | ***0.011*** | ***1.05*** |
| Proportion smoker (%) | - | - | *-* | *-* | - | - | *-* | *-* |
| ***Pandemic-related factors*** |  |  |  |  |  |  |  |  |
| Time since 1^st^ case (days) | -0.0080 | 0.0093 | *0.393* | *0.99* | -0.0154 | 0.0102 | *0.137* | *0.98* |
| Time since 100 cases (days) | - | - | *-* | *-* | 0.0250 | 0.0069 | ***0.001*** | ***1.03*** |
| Time since 1st death (days) | - | - | *-* | *-* | 0.0202 | 0.0126 | *0.114* | *1.02* |
| Testing coverage (n. tests per 10,000 pop) | -0.0279 | 0.0137 | ***0.046*** | ***0.97*** | -0.0220 | 0.0105 | ***0.042*** | ***0.98*** |
| ***Health system strength*** |  |  |  |  |  |  |  |  |
| Healthcare workers (n. per 1,000 pop) | 0.0119 | 0.0568 | *0.835* | *1.01* | - | - | *-* | *-* |
| Hospital beds (n. per 1,000 pop) | - | - | *-* | *-* | - | - | *-* | *-* |
| Health expenditure (% of GDP) | - | - | *-* | *-* | 0.2235 | 0.0618 | ***0.001*** | ***1.25*** |
| ***Cultural characteristics*** |  |  |  |  |  |  |  |  |
| Individualism vs. collectivism | 0.0022 | 0.0103 | *0.827* | *1.00* | -0.0247 | 0.0074 | ***0.002*** | ***0.98*** |
| Uncertainty avoidance | 0.0248 | 0.0092 | ***0.010*** | ***1.03*** | - | - | *-* | *-* |
| Indulgence vs. restraint | - | - | *-* | *-* | - | - | *-* | *-* |
| Long-term vs. short-term orientation | - | - | *-* | *-* | 0.0102 | 0.0071 | *0.157* | *1.01* |
| Power distance | - | - | *-* | *-* | - | - | *-* | *-* |
| Masculinity vs. femininity | - | - | *-* | *-* | - | - | *-* | *-* |
| **Political characteristics** |  |  |  |  |  |  |  |  |
| Polity (democracy vs authoritarianism) | 0.0639 | 0.0414 | *0.128* | *1.07* | 0.0543 | 0.0330 | *0.107* | *1.06* |
|  | pseudo-R^2^: 30 % | | | | pseudo R^2^: 58% | | | |
|  | AIC:202.5.9 BIC:225.1 | | | | AIC:169.5 BIC: 199.4 | | | |

Table 5. Random-effects meta-regression analysis of the crude case fatality risk at the last follow-up date (September 20, 2020) for 68 countries following the removal of statistical outliers. Dependent variables were logit transformation to stabilize the variance of proportions. Random-effects meta-regression was used to explore the impact of cultural characteristics on crude test positivity while adjusting for important predefined covariates following the removal of influential observations from the analysis in Table 2. Bold font indicates a statistically significant association with outcome at p<0.05.
